# Targeting Bronchopulmonary Dysplasia-Associated Pulmonary Hypertension (BPD-PH): Potential Role of the FGF Signaling Pathway in the Development of the Pulmonary Vascular System †

**DOI:** 10.3390/cells9081875

**Published:** 2020-08-11

**Authors:** Cho-Ming Chao, Lei Chong, Xuran Chu, Amit Shrestha, Judith Behnke, Harald Ehrhardt, Jinsan Zhang, Chengshui Chen, Saverio Bellusci

**Affiliations:** 1 Key Laboratory of Interventional Pulmonology of Zhejiang Province, Department of Pulmonary and Critical Care Medicine, The First Affiliated Hospital of Wenzhou Medical University, Wenzhou 325035, China; zhang_jinsan@163.com (J.Z.); wzchencs@163.com (C.C.); 2 Cardio-Pulmonary Institute, Universities of Giessen and Marburg Lung Center, Member of the German Center for Lung Research, Justus-Liebig-University Giessen, 35392 Giessen, Germany; xuran.chu@mail.utoronto.ca (X.C.); amshre@hotmail.com (A.S.); 3 Department of General Pediatrics and Neonatology, Justus-Liebig-University, Feulgenstrasse 12, D-35392 Gießen, Universities of Gießen and Marburg Lung Center, German Center for Lung Research, 35392 Giessen, Germany; judith.behnke@paediat.med.uni-giessen.de (J.B.); harald.ehrhardt@paediat.med.uni-giessen.de (H.E.); 4 Institute of Pediatrics, National Key Clinical Specialty of Pediatric Respiratory Medicine, Discipline of Pediatric Respiratory Medicine, The Second Affiliated Hospital of Wenzhou Medical University, Wenzhou 325027, China; chongpeilei@gmail.com; 5 School of Pharmaceutical Sciences, Wenzhou Medical University, Wenzhou 325035, China; 6 International Collaborative Center on Growth Factor Research, Life Science Institute, Wenzhou University, Wenzhou 325035, China

**Keywords:** Bronchopulmonary dysplasia, pulmonary hypertension, FGF signaling

## Abstract

More than 50 years after the first description of Bronchopulmonary dysplasia (BPD) by Northway, this chronic lung disease affecting many preterm infants is still poorly understood. Additonally, approximately 40% of preterm infants suffering from severe BPD also suffer from Bronchopulmonary dysplasia-associated pulmonary hypertension (BPD-PH), leading to a significant increase in total morbidity and mortality. Until today, there is no curative therapy for both BPD and BPD-PH available. It has become increasingly evident that growth factors are playing a central role in normal and pathologic development of the pulmonary vasculature. Thus, this review aims to summarize the recent evidence in our understanding of BPD-PH from a basic scientific point of view, focusing on the potential role of Fibroblast Growth Factor (FGF)/FGF10 signaling pathway contributing to disease development, progression and resolution.

## 1. Introduction

There is a rising incidence of preterm births which is one of the leading cause of death in infants younger than 5 years of age [1,2]. Bronchopulmonary dysplasia (BPD) is a common complication for infants born before 30 weeks of gestational age. The incidence of BPD is approximately 40%. BPD contributes substantially to long-term morbidities and mortalities [3]. Currently, there is no curative therapy for BPD.

BPD is a complex chronic lung disease caused by the interplay of lung development and pre- and postnatal injurious events such as ventilatory damages, oxygen toxicity, infectious stimuli, growth restriction, and repair/remodeling processes. Due to advances in the clinical management of preterm infants (e.g., use of exogenous surfactant, antenatal administration of steroids, more gentle non-invasive ventilation, and nutritional strategies in the clinical management of preterm infants) the mortality has reduced but the proportion of very early preterm infants has increased. During the so-called post-surfactant era, remarkable changes regarding the histomorphology of fatal BPD have been observed. The ”new“ BPD is characterized by simplification of alveolar formation and arrested microvascular development as compared with fibroproliferative processes mostly of the airways in ”old“ BPD. Whether these histomorphological changes result from different windows of injury (very early stages versus late stages of lung development) or from different treatment approaches (e.g., harsh mechanical ventilation with high oxygen versus non-invasive ventilation) is still unclear.

Histomorphologically, BPD is mainly characterized as alveolar simplification and pulmonary vascular remodeling. Pre- and postnatal pulmonary inflammatory responses (e.g., chorioamnionitis) are central risk factors for developing BPD. Pulmonary inflammatory responses can be induced by oxygen therapy or mechanical ventilation leading to the imbalance of proinflammatory and anti-inflammatory cytokines. This is associated with an accumulation of inflammatory cells in the lung. For example, it has been demonstrated that interleukin 10 (IL-10) and growth factors such as vascular endothelial growth factor alpha (VEGFA), platelet derived growth factor alpha (PDGFA), and fibroblast growth factors (FGFs) are decreased in tracheal aspirates and lung tissue [4,5,6]. 

The risk of developing BPD correlates inversely with birth weight. Research studies on intrauterine growth restriction (IUGR) have confirmed that premature newborns have a higher risk of suffering from BPD due to significant changes in the metabolic, hormonal, and hemodynamic status [7,8,9]. IUGR has been shown to disrupt developmental pathways, for example, epithelial and endothelial cell differentiation of the lungs leading to decreased alveolar and vascular growth [10]. One known mechanism is the downregulation of VEGF, a proangiogenic factor. Furthermore, IUGR has been shown to decrease elastin expression and deposition in rat lungs resulting in altered lung function associated with diminished lung compliance [11]. A recent study by Blair Dodson and colleagues demonstrated that IUGR altered the nuclear factor “kappa-light-chain-enhancer” of activated B-cells (NF-κB) pathway, which has been known to promote pulmonary vascular growth during lung development. They showed decreased NF-κB signaling in pulmonary artery endothelial cells (PAECs) using a sheep model for IUGR (chronic placental insufficiency). Furthermore, remarkable alterations in the expression of extracellular matrix (ECM) pathways, including decreased collagen and laminin, have been detected using microarray analysis [12].

Here, we also refer to other excellent reviews for more detailed information on our recent understanding of the pathogenesis of BPD [13,14]. Apart from the abovementioned advances, the use of exogenous surfactant, antenatal administration of steroids, more gentle non-invasive ventilation, and nutritional strategies in the clinical management of preterm infants, which collectively started within the last decades, there are no new effective targeted treatment concepts to prevent and treat BPD [15]. Furthermore, the definition of BPD that describes the infants at risk of BPD or that predicts long-term adverse outcomes such as pulmonary hypertension, is currently a matter of discussion [16].

As mentioned above, in addition to the disturbed alveolarization, dysmorphic pulmonary vasculature is a key pathologic feature in BPD, leading to abnormal hemodynamic and cardiac mechanic resulting in devastating pulmonary hypertension (PH) [17]. For easier readability, thereafter, we are using the term BPD-PH for BPD-associated PH. 

In order to develop new approaches to treat BPD-PH, we need to understand the mechanisms underlying the normal development of alveoli and associated pulmonary vessels and how these mechanisms are impaired under disease conditions.

Over the past two decades, a growing number of publications have revised the existing paradigm that the development of pulmonary vessels passively follows the development of airways and alveoli. Indeed, an active leading role of the vasculature in alveologenesis has been proposed. In fact, supporting evidence comes from loss-of-function experiments in diverse animal models which have shown that inhibition of vascular endothelial growth factor (Vegf) leads to decreased alveolarization [18,19,20,21].

In addition to disrupted alveolarization, Lazarus et al. have shown that the vasculature was essential for epithelial airway branching during development. Different ablation strategies have been used to eliminate lung blood vessels to demonstrate the contribution of blood vessels to branching stereotypy of mouse lung airways. One of the methods consisted of the conditional inhibition of Vegf protein activity in the mouse lung. *Surfactant protein c (Sftpc)-rtTA* transgenic mice, which is a lung epithelial specific driver line, were crossed with a responder line, which is composed of a doxycycline-inducible IgG1-Fc tail fused to the extracellular domain of Vegfr1. The results showed that early in lung development, conditional inhibition of Vegf activity led to vascular defects without adverse effects on the epithelium and mesenchyme. However, the reduction of the vasculature in vivo led to fewer and dilated airway branches that were rescued upon re-vascularization. Transient blockade of Vegf-mediated angiogenesis from E6.5 until E12.5 and examination of the lungs at E14.5 indicated that, compared with the pups that underwent continuous Vegf blockade (from E9.5 to E14.5) and the control littermates, the pups that underwent transient Vegf inhibition resumed vascularization. The regain of the vascular network (quantified by processing digital images with the aid of ImageJ software calculating the number of double-positive cells per an epithelial surface area unit after double immunostaining with pH3 and E-Cadherin) was almost rescued by E14.5, accompanied by partial resumption of lung branching [22] 

Accordingly, gain-of-function of Vegf enhanced alveolarization after hyperoxic lung injury in rodent BPD models [19,23,24,25]. In line with these preclinical findings, angiogenic factors such as VEGF and its associated receptor FMS-like tyrosine kinase (FLT-1) (encoding VEGFR1), as well as the angiopoetin-1 receptor (also called TIE-2) have been reported to be crucial players both during normal pulmonary vascular formation and in human preterm infants suffering from BPD [4,26,27,28,29,30,31]. Despite these paradigm-changing findings, the intricate mechanisms orchestrating endothelial cell proliferation, maturation, and migration during normal pulmonary vascular formation, as well as survival and regeneration during vascular remodeling (which refers to changes in the size, shape, structure, and function of the vessel leading to increased pressure in the pulmonary circulation) and reverse remodeling (a process allowing the restoration of the normal vascular function), are incompletely understood. All the underlying disruptive events are most likely attributable to the development of PH in BPD.

Pulmonary hypertension (PH) is a common and life-threatening complication in BPD. A recent meta-analysis including 1400 preterm infants revealed that the prevalence of PH increases correspondingly with the severity of BPD. The prevalence is estimated to be approximately 40% in preterm infants suffering from severe BPD [32,33]. 

Pulmonary vascular remodeling (VR) underlying PH leads to (I) increased muscularization of already muscularized proximal and middle-sized vessels due to proliferation of existing vascular smooth muscle cells (VSMCs) and (II) *de novo* muscularization of normally non-muscularized distal vessels. The latter are typically the distal arterioles with diameters under 30 µm. VR results in increased pressure in the pulmonary circulation leading to an increased right ventricle (RV) afterload and potentially fatal RV hypertrophy (RVH) [34,35,36]. It needs to be mentioned that the cellular origin of the newly formed VSMCs in BPD is still elusive. The complications associated with BPD without PH includes a higher risk for developing susceptibility to infections of the upper and lower respiratory tract and asthma. By contrast, infants suffering from BPD-PH are of higher risk for oxygen dependency and right ventricle hypertrophy, which requires long-term medication and follow-up. In the worst case, these additional complications can lead to right heart failure. The lifelong morbidity of patients suffering from BPD-PH represents a substantial and growing burden for patients and health care systems [37,38].

Although recent guidelines published by the American Heart Association (AHA), American Thoracic Society (ATS), the European Pediatric Pulmonary Vascular Disease Network (PVD), and the Pediatric Pulmonary Hypertension Network (PPHNet) encourage and improve the implementation of standardized diagnostic and treatment protocols, the molecular and cellular pathomechanisms underlying BPD-associated PH remain poorly characterized, reflected by the rather limited pharmacotherapeutic options available to mitigate but not cure BPD-PH [39,40,41] 

This review aims to summarize the recent progress in our understanding of BPD-PH from a basic scientific point-of-view, focusing on the potential role of fibroblast growth factor (FGF)/FGF10 signaling pathway contributing to disease development, progression, and resolution. 

## 2. Development of Normal Pulmonary Vasculature and BPD-PH from the Basic Scientific Point-of-View

According to the Fifth World Symposium held in France, PH is clinically classified into five groups (Group 1, pulmonary arterial hypertension (PAH); Group 2, pulmonary hypertension due to left heart disease; Group 3, pulmonary hypertension due to chronic lung disease or hypoxia; Group 4, chronic thromboembolic pulmonary hypertension; and Group 5, pulmonary hypertension due to unclear multifactorial mechanisms) [42,43]. Groups 1, 3 and 4 are all defined as precapillary pulmonary hypertension [44]. Therefore, they share similar molecular and cellular mechanisms and histopathology. Pediatric PH has some common features with adult PH but also shows its own features. BPD-PH, characterized by impaired alveolar growth and distorted pulmonary vascular development, is categorized as a subgroup of Group 3 (Group 3.5, developmental lung disorders) [45]. Therefore, in the next section, we describe the different lung vascular growth abnormalities and the potential reasons of BPD-PH from the developmental angle.

### 2.1. Normal Development of Pulmonary Vasculature

#### 2.1.1. Lung Vasculature Formation and Maturation

Human lung development can be histologically divided into four distinguishable stages: embryonic and pseudoglandular stages (human, week 4–17 and mouse, E9.5–E16.5), canalicular stage (human, week 17–26 and mouse, E16.5–E17.5), saccular stage (human, week 26–36 and mouse, E17.5–PN5), and alveolar stage (human, week 36–8 years and mouse, PN5–PN30) [46,47].

The vasculature starts to develop as early as the lung buds evaginate from the foregut endoderm [48]. At E10, angioblasts and hematopoietic cells, localized at the distal mouse lung buds, form the blood islands through a processed termed vasculogenesis. The blood islands are comprised of circumferential layers of flattened angioblasts which represent primitive endothelial cells (ECs) and inner hematopoietic cells [49]. From E11 to E12, proximal vessels sprout from the main pulmonary trunk (proximal angiogenesis), run along the main conducting airways, as well as its many ramified branches. In addition, the distal blood islands increase markedly and are connected together to form the primitive capillary plexus (distal angiogenesis) [46]. During the late pseudoglandular stage, the capillary network, around lung buds, fuses with the proximal vessels. This capillary network expands significantly during the canalicular and saccular stages and gradually gets close to each other to become a double capillary layer between the adjacent lateral walls of distal sacs which form during the process of primary septa formation. During alveologenesis, concomitant with the formation of the secondary septa, the capillary network folds up to form new double capillary layers. These double capillary layers further evolve into a single capillary layer through a process called microvascular maturation. This process reduces the distance between the alveolar walls and the capillaries, thereby facilitating efficient gas exchange [50,51]. Microvascular maturation occurs concomitantly to alveologenesis and, in humans, lasts until young adulthood [52]. 

#### 2.1.2. Pulmonary Vascular Development Needs Multicellular Crosstalks

Interactions among different cell types are indispensable for the formation and maturation of the vascular system during lung development. At the early stage of lung development, mesenchymal cells express Vegf, which interacts with its receptors, Vegfr1 (Flt-1), Vegfr2 (Flk-1), and Vegfr3 on endothelial cells (ECs), initiating and regulating the formation of vasculogenesis and angiogenesis [53,54]. Vegfr2 is the most important signaling receptor, which upon activation, initiates and promotes vasculogenesis and angiogenesis [55]. In contrast, Vegfr1 functions as a ligand trap, therefore, reducing the interaction between Vegf and Vegfr2, thereby decreasing the overexpansion of ECs [56]. From the late pseudoglandular stage to the canalicular stage, the cellular source of Vegf gradually shifts from the resident mesenchymal cells to the alveolar epithelial cells (mainly alveolar type 2 (AT2) cells). This results in further attraction of the capillaries towards the epithelium, thereby promoting the formation of primitive alveolar septa through EC-derived angiocrine factors, such as hepatocyte growth factor (Hgf) [57,58,59]. 

**Figure 1:**
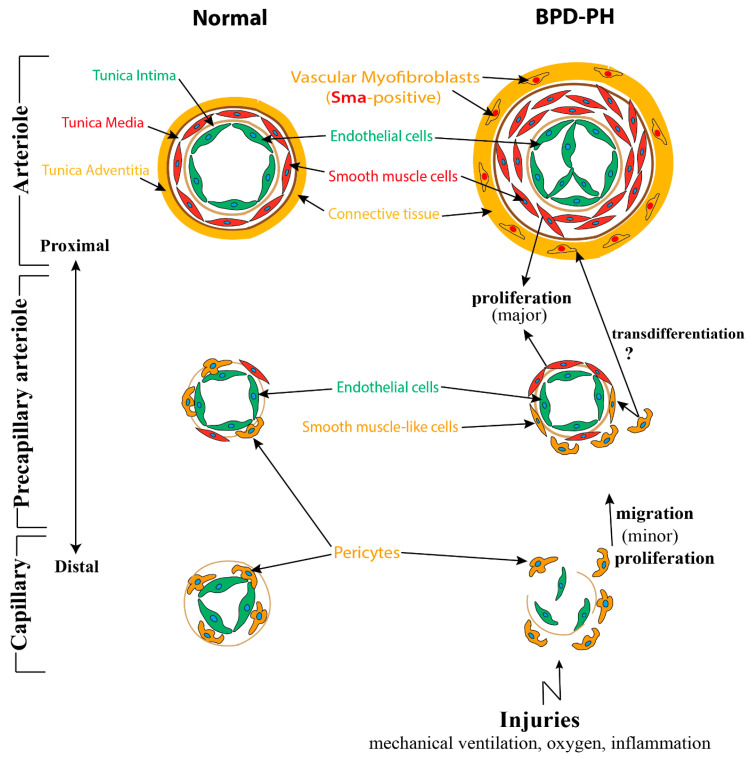
A speculative model of structural changes of the pulmonary vessel wall in bronchopulmonary dysplasia-associated pulmonary hypertension (BPD-PH). Under a normal situation, pulmonary arterioles are wrapped by the following three layers of tunicae: The tunica intima consists of endothelium, basement membrane, and internal elastic tissue; the tunica media is comprised of smooth muscle cells and external elastic tissue; and connective tissues constitute the tunica adventitia. From large vessels to small vessels, tunica media and adventitia are gradually getting sparse and the smooth muscle cells gradually evolving into pericytes, which adhere tightly to capillary endothelial cells (ECs). While in BPD, due to multifactorial injuries, the endothelial cells are dysfunctional and eventually become apoptosis resistant. Smooth muscle cells, which mostly proliferated from resident smooth muscle cells (SMCs) are increased significantly, resulting in the thickening of tunica media and the muscularization of normally non-muscular vessels. Adventitia of pulmonary arterioles undergo α-Sma positive myofibroblast transition assisted by altered extracellular matrix breakdown and deposition. Pericytes disconnect with capillary ECs, leading to the loss of small capillaries, proliferate, and migrate into the mesenchyme and also contribute to a small population of smooth muscle-like cells.

Following the interaction between epithelium and mesenchyme, a population of mesenchymal cells differentiate into α-smooth muscle actin (Sma)-positive mural cells (VSMCs and pericytes), another component of the blood vessel wall [60]. VSMCs, which display a flattened, spindle, and dense structure, are usually present in the media of large vessels such as arteries, arterioles, and veins. From large vessels to capillaries, VSMCs are gradually getting sparse and changing into round, protruded cells, termed pericytes. They always embed in the basement membrane and adhere tightly to ECs [61,62]. (Figure 1). In contrast to VSMCs, several markers including neuron-glial antigen-2 (Ng2), Cd146, α-Sma, Sm22, desmin, platelet-derived growth factor receptor-β (Pdgfr-β), aminopeptidase A and N, RGS5, and the promoter trap transgene XlacZ4 have been identified to label pericytes [62,63,64,65].

Pericytes play a critical role in regulating microvascular and alveolar development. Pericytes, on the one hand, adhere to capillary ECs to regulate their function, proliferation, migration, and differentiation via several signaling pathways including Vegf/Vegfr, Pdgfb/Pdgfrb2, Tgfβ/Alk, S1p/Endoglin 1(Edg-1), Ang1/2/Tie2, Cadherin, and Notch signaling pathways [51,66,67,68,69,70]. On the other hand, pericytes also interact with AT2 cells to promote secondary septa formation through the Hippo pathway components Yap and Taz [71]. In addition, pericytes can also function as pulmonary stem cells, upon stimulation, transdifferentiating into VSMCs and myofibroblasts [72,73].

Interactions between endothelial cells (ECs) and smooth muscle cells (SMCs) play a major role in pulmonary vasculature. Normal interplay among these two cell types control the homeostasis of the pulmonary circulation, whereas aberrant association can contribute to the pulmonary vascular diseases and pulmonary hypertension. It has been well established that the release of various vasoactive agents such as nitric oxide (NO) and endothelin-1 (Et−1) through paracrine signaling endothelial cells regulates smooth muscle cell activity. However, other non-paracrine signaling mediated crosstalk between EC and SMC (such as communication through myoendothelial junctions, as well as interaction via extracellular vesicles) exists, which is altered under the pathological condition, thus, causing an increase in vasocontractility and abnormal vascular proliferation, therefore, leading to vascular remodeling, right ventricular hypertrophy, and pulmonary hypertension [74]. 

### 2.2. Pathologic Development of Pulmonary Vasculature Leading to PH in BPD

#### 2.2.1. Abnormalities of Pulmonary Vasculature Observed in BPD-PH

Impaired pulmonary vascular development, VR and PH are likely associated with interruption of vascular formation during lung development. However, not all premature infants develop BPD or BPD-PH. It has been claimed that genetic components (e.g., polymorphisms in matrix metallopeptidase 16 (*MMP16*) and SPARC (Osteonectin) Cwcv and Kazal like domains proteoglycan 2 (*SPOCK2*) could play a role regarding the susceptibility for BPD [75].

We previously found that conditional deletion of phosphatase and tensin homologue (*Pten*) in early embryonic mouse lung mesenchyme led to lethality at birth with disorganized alveolar capillary beds [76]. This phenotype is similar to the lethal alveolar capillary dysplasia phenotype observed in newborn babies. In support of developmental perturbations, BPD often occurs in preterm infants born before the alveolar stage, and thus susceptible to various injuries such as mechanical ventilation, hyperoxia, and airway inflammation. These, in turn, affect pulmonary vascular development, leading to vascular remodeling, PH, and impaired alveologenesis [77,78].

**Figure 2:**
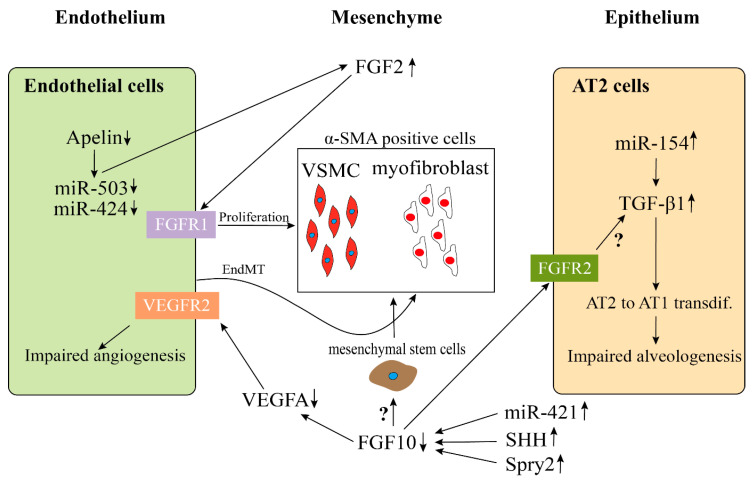
Possible endothelial–mesenchymal and epithelial–mesenchymal interactions in BPD-PH. Disrupted endothelial Apelin, *miR-503*, and *miR-424* results in an increased expression of FGF2 and induces the hyperproliferation of vessel smooth muscle cells (VSMC). An increase of α-SMA-positive myofibroblasts could be due to endothelial-mesenchymal transition (EndMT). The continuous high expression of *miR-154* in AT2 cells stimulate the activation of TGFβ1 signaling pathway, which results in an impaired alveologenesis. Decreased FGF10 expression in BPD, which is caused by the upregulation of *miR-421*, SHH, and Spry2, leads to the downregulation of VEGFA and upregulation of α-SMA, ultimately resulting in an impaired angiogenesis and an increase of α-SMA positive cells. However, whether FGF10 acts directly on the mesenchymal stem cells (MSC) to induce them to differentiate into α-SMA positive cells or through activating TGFβ1 signaling pathway needs to be further investigated.

Bhatt et al. analyzed lung samples from infants who died with BPD versus infants who died from non-pulmonary causes and found a decreased expression of VEGF and platelet endothelial cell adhesion molecule-1 (PECAM, also termed CD31, endothelial marker), as well as a decreased staining density of alveolar capillaries in BPD infants, indicating that the development of the pulmonary microvasculature was disrupted in BPD patients [4]. Consistent with previous results, we and others, utilizing BPD animal models, established by hyperoxia exposure, also showed a decrease of endothelial cells in capillaries and blood vessel numbers and an increase of α-Sma positive cells (VSMC) in the tunica media of pulmonary arterioles and normally non-muscularized precapillary arterioles [79,80,81]. A lineage tracing study indicated that the expansion of resident SMCs was the major source related to the thickening of the smooth muscle layer in adult PH [82]. An increased collagen and elastin expression associated with increased α-SMA-positive myofibroblasts, possibly due to endothelial to mesenchymal transition (EndMT), has also been proposed as a mechanism for the accumulation of myofibroblats in the adventitia of pulmonary arteries (Figure 2) [83,84].

In addition, it has also been proposed that the pericytes play a potential role in the microvascular remodeling of PH. In a clinical study, Assaad et al. found that the numbers of pericytes associated with upregulated PDGF-B expression were significantly increased in pulmonary capillary hemangiomatosis (PCH) patients, a cause of PH [85]. Furthermore, Ricard et al. also showed an increased migration and proliferation of pericytes induced by FGF2 and IL-2, in human PH patients. Finally, increased TGFβ also promotes the transdifferentiation of pericytes into contractile α-SMA positive cells (Figure 1) [86]. 

#### 2.2.2. Impaired Multicellular Interactions Disrupt Pulmonary Vascular Development in BPD-PH

Multiple studies aiming at elucidating the pathogenesis of pulmonary VR have demonstrated an impairment of multicellular interactions, which could have resulted from abnormal expression levels of signaling pathways involved in cell–cell interactions.

As aforementioned, the Vegf signaling pathway is one of the most important signaling pathways regulating epithelial-endothelial crosstalk during pulmonary vascular development. Multiple studies have demonstrated that Vegf and Vegfr2 expressions were significantly decreased, while the expression of soluble Fms-like tyrosine kinase 1 (sFlt-1), an endogenous antagonist to Vegf corresponding to a truncated form of the Vegf receptor acting as a dominant negative Vegf receptor, was significantly increased, in experimental BPD animal models, which lead to a reduction and disarrangement of the microvasculature [87,88].

Other signaling pathways potentially involved in the angiogenic network include sonic hedgehog (Shh) and Sprouty2 (Spry2). Sprouty2 is an inhibitor of Fgf10. Fgf10 is known to be a major regulator of epithelial branching. Inhibition of Vegfr1-mediated signaling leads to upregulation of Spry2 in the epithelium, suggesting a downregulation of Fgf10 signaling. This is an essential evidence demonstrating the importance of endothelial-mesenchymal crosstalk. More evidence confirming the endothelial-epithelial crosstalk came from DeLisser and colleagues. *Pecam1*-deficient mice revealed disrupted endothelial cell formation associated with decreased alveolar simplification [22,89].

Furthermore, a recent study found a reduction of WNT5 in the pulmonary microvascular endothelial cells (PMVECs) of PH patients. Through exposing *Wnt5a* Endothelial cKO mice with chronic hypoxia, the authors demonstrated that loss of endothelium-derived Wnt5a impaired the endothelium–pericytes interaction, resulting in significant reduction, muscularization, and decreased pericyte coverage of microvessels [72]. Another study, conducted in adult PH, revealed that PH pericytes overexpressed C-X-C motif chemokine receptor-7 (CXCR-7) and TGFβRII, and as compared with control pericytes, they were more likely to proliferate, migrate, and differentiate into smooth muscle-like cells, indicating a significant role of endothelium–pericytes interaction in the process of PH (Figure 1) [90].

Pericyte-myofibroblast transition (PMT) could represent another pathogenesis of pulmonary VR, consistent with the results conducted in kidney fibrosis [91]. Wang et al. found that lung pericytes differentiated into myofibroblast in idiopathic pulmonary fibrosis (IPF) patients through increasing NOTCH1/PDGFRβ/ROCK1 signaling pathway [92]. However, the exact role of pericytes in the pathogenesis of BPD-PH is still unclear and needs to be further explored.

PDGFA/PDGFRA is an important signaling pathway controlling the elastogenesis of secondary crest myofibroblast during alveologenesis [93]. Interestingly, reduced PDGFRA has been found both in BPD patients and in hyperoxia-induced BPD mouse model [5,94]. Moreover, Oak and colleagues found that attenuated PDGFRA expression due to hyperoxia exposure resulted in a decrease of VEGFA, which ultimately led to increased ECs apoptosis and reduced microvessel numbers [94].

It is important to notice that FGF10 signaling has a close interaction with the VEGF signaling pathway. Therefore, we propose that FGF10 signaling plays critical roles in the pathogenesis of BPD-PH (discussed in detail in Section 3 below).

### 2.3. MicroRNAs (miRs) May Be Associated with the Pathologic Vascular Development of BPD-PH

A growing number of evidences show that expression of numerous microRNAs (miRs), that normally regulate different signaling pathways mediating cellular crosstalk in lung development, are dysregulated in PH and BPD. Thompson and Lawrie summarized a list of miRs which had therapeutic effects on PH from hypoxia or monocrotaline (MCT)-induced PH in animal experiments [44]. Combining the differentially expressed miRs in BPD versus control with Thompson and Lawrie’s findings in PH, we identified several miRs which were all dysregulated and had the same changes in both BPD and PH (Table 1) [95,96]. These dysregulated miRs could be involved in BPD-PH.

Furthermore, there is evidence that miRs target FGF signaling to regulate proliferation. In the study by Chen et al., the authors showed that *miR-339* inhibited the proliferation of pulmonary artery smooth muscle cells (PASMCs) by targeting FGF signals [97]. By using EdU incorporation assay, they showed that *miR-339* inhibited the proliferation of PASMC. In addition, they found that *miR-339* inhibited FGF2-induced proliferation of PASMCs but had no effect on PDGF-BB-induced proliferation via functional analysis. Using a bioinformatics tool (Targetscan), they found that FRS2 was a potential target of *miR-399* to target FGF signaling. 

In disease condition, it has also been shown that the Apelin (APLN) and FGF2 pathways in pulmonary artery endothelial cells (PAECs) are regulated by *miR-424* and *miR-503*. In PH, this pathway is disrupted (Figure 2) [98]. 

Yuan et al. found that the expression of *miR-421* in mice exposed to hyperoxia-induced lung injury (mouse model of BPD) was much higher than that of room air control mice. However, the expression of *Fgf10* decreased significantly as compared with the control group [99]. As a result, inhibition of *miR-421* reduced bronchopulmonary dysplasia in mice by upregulating *Fgf10* (Figure 2).

## 3. Role of FGF/FGF10 Signaling in Pulmonary Vascular Formation

Fibroblast growth factor 10 (*Fgf10*) is one of the most important genes expressed during lung development. Extensive research has shown that it is expressed in the submesothelial mesenchyme [112,113]. After secretion, Fgf10 acts in a paracrine fashion mainly through the receptor Fgfr2b located on the lung epithelium [114]. More and more research findings which have mainly used preclinical mouse models for lung injury, have confirmed the role of Fgf10 in protecting and regenerating the lung tissue during and after lung injury, e.g., in lung fibrosis [115,116,117]. Our recent data emphasized, that Fgf10 indeed plays a crucial role in branching morphogenesis and formation of the pulmonary vasculature during development [79,118]. As previously mentioned, the temporal–spatial proximity of the lung vasculature and the distal epithelium suggests a close interaction between these two important structures via endothelial–epithelial tissue crosstalk. Thus, it has been shown that mesenchymally secreted Fgf10 leads to the upregulation of Vegf in the distal epithelium [119]. Also supporting this hypothesis, we demonstrated, fifteen years ago, that treatment of embryonic lung explants with recombinant vascular endothelial growth factor A (Vegfa) not only upregulates Vegfr2 in the mesenchyme but also induces branching of the epithelium [120]. This finding underscores the notion, that branching morphogenesis and pulmonary vascular formation occur in a highly coordinated fashion. Interestingly, isolated lung epithelium treated in vitro with Vegf does not respond by branching indicating that the effect of Vegfa on epithelial branching is likely mediated via its action on the mesenchyme. This endothelial–epithelial tissue crosstalk has been extensively investigated by using in vitro studies co-culturing epithelium with the mesenchyme as well as by in vivo lung agenesis model [48]. By inducing the expression of a soluble dominant negative receptor of Vegfr1, it has been shown that Sprouty2 (Spry2), a negative regulator of tyrosine kinase signaling, is upregulated in the epithelium. This suggests inhibition of Fgf signaling [22]. In addition, mice owning a hypomorphic allele for *Fgf10* display decreased expression of *Vegfa* and *Pecam* resulting in simplified lung structure accompanied by an abnormally developed lung vasculature [121].

Our follow-up study on these *Fgf10* hypomorphic mice showed that *Fgf10* heterozygous newborn mice exposed to hyperoxia (85% hyperoxia, P0–P8) displayed a BPD-like lung phenotype with increased hypoalveolarization accompanied with decreased number of AT2 but an increased number of ATI. Further, transcriptomic analysis of isolated AT2 cells from mutant vs. control lungs revealed downregulated AT2 but upregulated AT1 gene signature in mutant AT2 cells associated with decreased surfactant protein b and c production. AT2 cells could, therefore, be both quantitatively and qualitatively dysfunctional in *Fgf10* hypomorphic lungs and it is possible that this could affect the proper formation of the pulmonary vasculature leading to PH in BPD [118].

Intriguingly, investigations on lung tissue of preterm infants that died from severe BPD revealed decreased *FGF10* expression [122]. While the direct effect of FGF10 on the lung endothelium still needs to be proven, the response of endothelial cells (ECs) to FGF signaling has been well described in vitro [123,124]. It has been shown by gene expression analysis that *FGFR1* and *FGFR2* are the major FGF receptors being expressed by ECs while *FGFR3* seems to play a minor role [125,126]. *FGFR4* was not found to be expressed in ECs [127]. The functional role of FGF signaling has been demonstrated by using a dominant negative receptor of all FGFRs (FGFR1DN). Murakami et al. showed that FGF signaling was important for homeostasis and vascular integrity [128,129,130]. The same authors showed that FGF signaling acted upstream of VEGF signaling in normal and disease conditions [127,129]. In order to test the cell-specific role of *FGFR1/2* in ECs, Oladipupo and colleagues used genetically modified mice to delete *FGFR1/2* in *Flk1* and *Tie2* expressing cells [131]. Both are cell markers for endothelial and hematopoietic cells. They showed that the genetic deletion of *Fgfr1/2* in endothelial cells did not lead to disruption of embryonic vascular development. In the adult mutant mice, the vascular integrity was maintained in the homeostatic condition. However, in injury models of eye and skin injury, the neovascularization process was significantly impaired and associated with delayed wound healing. Further evidence for the importance of Fgfr1/2 response in ECs upon injury was brought by House and colleagues. Again, using the *Tie2-Cre; Fgfr1^flox/flox^; Fgfr2^flox/flox^* mice, they demonstrated a worsened cardiac function (decreased vessel density, increased endothelial cell apoptosis, and hypokinetic areas) following cardiac ischemia-reperfusion injury [132]. The ligand acting on the endothelial cells via Fgfr1 and Fgfr2 is still unknown. Whether Fgf10 is acting directly on the endothelium or on the VSMCs still needs to be tested.

It has been shown in mice that Fgf10-positive cells were progenitors for VSMCs in vivo during lung development. The genetically modified mice displaying a *knock in* of CreERT2 frame with the first exon of Fgf10 crossed with the tdTomato reporter mice (Fgf10^iCre/+^; tomato^flox/+^) were used. These mice allowed us to permanently label Fgf10-positive cells following tamoxifen treatment. Single IP injection of tamoxifen was carried out at E11.5 or E15.5 and embryonic lungs were harvested at E18.5. Two waves of Fgf10 expression during embryonic lung development were observed. In the first wave, the Fgf10-positive cells residing in the submesothelial mesenchyme (and their progeny) contributed to the formation of airway smooth muscle cells (ASMCs), VSMCs, and lipofibroblasts at E18.5. Interestingly, the second wave of Fgf10-positive cells labeled at E15.5 did not give rise to VSMCs [133]. Several studies have suggested that the abnormal accumulation of SMCs in the distal pulmonary arterioles leading to its excessive muscularization was a major contributing factor to the pathology of PH. In a mouse model of hypoxia-induced PH, studies have shown that a significant amount of distal arteriole SMCs were derived from pre-existing smooth muscle cells [34]. It could be possible that these SMCs arising from Fgf10-positive cells during early development, could be one of the key cell types responsible for the increased muscularization of distal arterioles during hypoxia-induced PH. However, this needs further confirmation.

In a previous but nevertheless related study, *Mlc1v-nLacZ-24* transgenic mice (thereafter called *Fgf10^LacZ^*), in which the expression of LacZ was controlled by Fgf10 regulatory sequences, were used to monitor the localization of Fgf10-positive cells. By using whole-mount in-situ hybridization and X-gal staining on the E12.5 and E13.5 embryonic lungs, it was found that β-galactosidase-positive cells were present exclusively in the distal mesenchyme, as well as adjacent to the bronchial epithelium. In addition, these β-galactosidase-positive cells around the bronchial epithelium also expressed the smooth muscle cell marker α-Sma suggesting that in the distal mesenchyme, the β-galactosidase-positive cells (Fgf10-positive cells) were progenitors for ASMCs. Reciprocal xenotransplantation on wild type and *Fgf10^LacZ/+^* embryos of the distal mesenchyme, as well as live imaging, demonstrated that β-galactosidase-positive cells in the distal mesenchyme were passively relocated around the bronchi [113].

In further studies, it was demonstrated that the *Mlc1v-nLacZ-24* allele behaved as an hypomorphic *Fgf10* allele opening the way to generate allelic series of mice displaying different levels of Fgf10 expression. These allelic series were generated by crossing the *Fgf10^LacZ^* transgenic line with *Fgf10^+/–^* mice on a C57Bl/6 background. Controls (*Fgf10^+/+^*) and *Fgf10^LacZ/–^* embryos were generated at different developmental stages.

By performing whole-mount immunohistochemistry with α-Sma antibodies in control and *Fgf10^LacZ/-^*, the authors found that the reduction in *Fgf10* expression led to decreased SMCs around bronchi, as well as a completely disorganized vasculature correlating with decreased Vegf expression [121]. 

Besides Fgf10 signaling, other Fgfs are also involved in the formation of the pulmonary vasculature. White et al. demonstrated that both fibroblast growth factor 9 (Fgf9) and sonic hedgehog (Shh) signaling to the pulmonary mesenchyme (and not to the endothelial cells) was necessary and sufficient for the development of distal capillaries. However, Fgf9 could only partially reverse the decreased capillary density found without Shh signaling. Shh could not rescue the vascular phenotype of *Fgf9* null lungs. These results suggest that Fgf9 and Shh signaling synergistically regulate the growth and pattern of the pulmonary capillary plexus and regulate the temporal and spatial expression of *Vegfa* [54]. Fgf9 and Shh regulate mesenchymal Vegfa expression and thereby, the development of the pulmonary capillary network.

## 4. Conclusions

In conclusion, BPD-PH is affecting a population of preterm infants which should not be neglected, with considerably high burdens for the patients and the health care system. On the basis of data mainly gained from preclinical research in mice, it has become increasingly evident that vascular remodeling (VR) is the pathological basis for pulmonary hypertension in BPD.

However, the origin of the newly formed VSMCs during VR (in both the proximal arteries and the more distal arterioles) and their fate during reverse remodeling is not yet understood. In the future, lineage tracing experiments in the context of experimental BPD in mice (using the Sugen inhibitor 5416 to block Vegf signaling) will be instrumental to characterize the progenitors for these newly formed VSMCs in the disease context. Targeting these progenitor cells could provide an opportunity to treat the disease in human patients.

The BPD mouse model is a well-established and widely accepted model to mimic the lung phenotype of BPD (alveolar simplification and dysmorphic lung vasculature) but in the context of BPD-PH, it needs to be mentioned that the only use of hyperoxia to induce lung injury is not causing PH in mice. A BPD-PH animal model needs to be established. It has been shown recently, that blockade of Fgfr2b ligands activity postnatally in a BPD mouse model lead to decreased blood vessel number and increased muscularization resembling a PH-like phenotype [79].

FGF10/FGFR2b signaling is believed to be essential in lung development. More interestingly, growing evidence coming from experimental research is confirming its preventive and regenerative effect during regeneration in lung diseases, such as BPD. In line with this notion, clinical-grade recombinant FGF10 could have therapeutic potential for the treatment of BPD-PH. However, based on the facts that other FGFs (e.g., FGF1 and FGF2) have been shown to be involved in the remodeling process of bronchial airways in COPD, it has to be taken into consideration that FGFs could be potentially detrimental. Therefore, the therapeutic use of FGF10 needs to be thoroughly validated [134]. In addition, potential adverse side effects need to be evaluated [135]. As a first translational step, the authors suggest the validation of the preventive and therapeutic effect of recombinant FGF10 in a mouse model of BPD-PH. Large animal models would allow the intratracheal or inhalative administration of recombinant FGF10 which would be much more favorable and clinically relevant.

Apart from the abovementioned translational approaches, FGF10 expression (both at the gene and protein levels) needs to be better clinically characterized within the heterogeneous cohort of preterm infants at risk of developing BPD-PH (e.g., gestation age, risk factors for BPD-PH, postnatal ventilation regimes, and genetic predisposition). Thus, future studies including the measurement, for example, using tracheal aspirates or exhaled breath condensate (EBC), of the expression of FGF10 in preterm infants is highly desirable. The establishment of a centralized biobank and databank would promote collection and characterization of biomaterials, provide organized availability and facilitate accessibility for research. In parallel to efforts invested in the clinical setting, more basic research needs to be done in order to gain deeper insight into the cellular and molecular mechanisms of FGF signaling governing vascular remodeling and reverse remodeling processes in BPD-PH.

The combination of both clinical and basic research would allow a better design of therapeutic strategies to prevent and to treat BPD-PH in the future.

## Figures and Tables

**Table 1:** MicroRNAs (miRs) potentially involved in pulmonary vascular remodeling in BPD.

miRs Shown to be Involved	Changes in PH and BPD	Potential Roles in BPD-PH	References
*miR-21*	increased	Increases PASMCs proliferation through inhibiting Bmpr2 activation	[100,101]
*miR-29*	increased	Impairs PASMCs and ECs function through down-regulating PPARγ	[102,103,104]
*miR-34a*	increased	increases lung epithelial cell apoptosis through down-regulating ANG1-TIE2 signaling; impairs alveologenesis through increasing PDGFR alpha-expressing myofibroblasts	[105,106]
*miR-126*	decreased	Decreases microvessel density through up-regulating SPRED-1	[101,107]
*miR-130*	increased	Promotes ECM remodeling, increases PASMCs proliferation and crosstalk through down-regulating PPARγ-APOE-LRP8 axis	[96,108]
*miR-154*	increased	Impairs alveologenesis through increasing TGFβ.	[109,110,111]
*miR-421*	increased	Impairs alveologenesis through down-regulation of FGF10 signaling	[99]
*miR-503*	decreased	Impairs ECs function and induces PASMCs proliferation through upregulating FGF2 and FGFR1	[96,98]

Abbreviations: PASMCs, pulmonary artery smooth muscle cells; ECs, endothelial cells; BMPR2, bone morphogenetic protein receptor type 2; PPARγ, peroxisome proliferator-activated receptor gamma; Ang, angiopoietin-1; PDGFRalpha, platelet-derived growth factor receptor alpha; SPRED-1, sprouty-related EVH1 domain containing 1; APOE, apolipoprotein E; LRP8, LDL receptor-related protein 8; TGFβ, transforming growth factor beta; FGF, fibroblast growth factor.
